# The complete chloroplast genome of an endangered endemic herb species in China, *Primula filchnerae* (Primulaceae)

**DOI:** 10.1080/23802359.2019.1644221

**Published:** 2019-07-24

**Authors:** Hua-Ying Sun, Li Zhong, Qi-Liang Gan, Ting Zhang, Zhi-Kun Wu

**Affiliations:** a Yunnan Key Laboratory for Dai and Yi Medicines, Yunnan University of Chinese Medicine, Kunming, China;; b Kunming Institute of Botany, Chinese Academy of Sciences, Kunming, China;; c Zhuxi Qiliang Institute of Biology, Zhuxi, China;; d Department of Pharmacy, Guizhou University of Traditional Chinese Medicine, Guiyang, China

**Keywords:** Complete chloroplast genome, phylogenetic analysis, *Primula filchnerae*

## Abstract

*Primula filchnerae* (Primulaceae) is an endangered endemic herb species in China. In this study, we characterized the complete chloroplast genome of *P. filchnerae* based on next generation sequencing (NGS). The chloroplast genome of *P. filchnerae* was 151,547 bp in size, containing a large single-copy (LSC) region of 82, 662 bp and a small single-copy (SSC) region of 17,749 bp. These two regions were separated by a pair of inverted repeat regions (IRs), each of 25,568 bp. A total of 130 functional genes were encoded, consisted of 86 protein-coding genes, 36 tRNA genes, and eight rRNA genes.

*Primula filchnerae* R. Knuth, belonging to sect. *Auganthus* of Primulaceae, is an endangered perennial herb species endemic to China (Hu 1990). It was first collected by Filchner in 1904 in Qinling Mountains, China. Since then, *P. filchnerae* was somewhat in mystery as it could not be resampled from the field (including its type locality) over 100 years, and the species was believed to be extinct. In 2006, two small wild populatons of *P. filchnerae* were rediscovered in Zhuxi County and Zhushan County, Hubei Province (Gan and Li 2015). The complete plastome could provide valuable genomic information for the conservation and restoration of this relict species.

Fresh leaves tissues of *P. filchnerae* were collected from a wild population (109°31′58″E, 32°19′20.49″N) in Zhuxi County. The voucher specimen was deposited in the herbaria of Kunming Institute of Botany (KUN 1444082). We isolated total genomic DNA following a modified CTAB protocol (Doyle 1991). The purified genomic DNA was sheared into *c*. 500 bp fragments to construct a paired-end (PE) library according to the Nextera XT sample preparation procedures (Illumina, San Diego, CA). We generated the PE reads of 150 bp using HiSeq X-Ten sequencer (Illumina, San Diego, California, USA).

In all, 3.3 Gb of raw sequence data were obtained. Reads were assembled into contigs using program CLC Genomics Workbench version 8.5.1 (CLC Inc, Arhus, Denmark). We annotated the complete plastome using the DOGMA pipeline (Wyman et al. 2004) and validated by comparing with the chloroplast genome of *P. sinensis* Sabine ex Lindl. (KU321892) (Liu et al. 2016). We determined transfer RNAs using tRNAscan-SE (Schattner et al. 2005), and a circular map of annotated genome was generated by using OGDRAW (http://ogdraw.mpimp-golm.mpg.de/) (Lohse et al. 2013). The annotated chloroplast genome of *P. filchnerae* was deposited into GenBank with the accession number MK888698.

The complete chloroplast genome of *P. filchnerae* was 151,547 bp in size with a typical quadripartite structure, containing a large single-copy (LSC) region of 82,662 bp and a small single-copy (SSC) region of 17,749 bp. These two regions were separated by a pair of inverted repeat regions (IRs), each of 25,568 bp. A total of 130 functional genes were encoded, consisted of 86 protein-coding genes (PCG), 36 tRNA genes, and eight ribosomal RNA (rRNA) genes. Of these genes, 18 genes were duplicated in the IR region, including seven protein-coding genes, seven tRNA genes, and four rRNA genes. Eighteen genes contain one or two introns. The overall GC content of the chloroplast genome was 37.2%, whereas the corresponding values of the LSC, SSC, and IR regions were 35.2, 30.5, and 42.8%, respectively.

Phylogenetic tree was reconstructed using RAxML (Stamatakis 2014) based on 11 complete chloroplast genomes of *Primula* and *Androsace laxa* as outgroup. The phylogenetic results indicated that the sampled *Primula* species clustered as a monophyletic clade with high support (100%), and *P. filchnerae* formed a clade with *P. sinensis*, with 100% bootstrap values (Figure 1). This published *P. filchnerae* chloroplast genome will provide useful information for phylogenetic and evolutionary studies in *Primula*.

**Figure 1.**
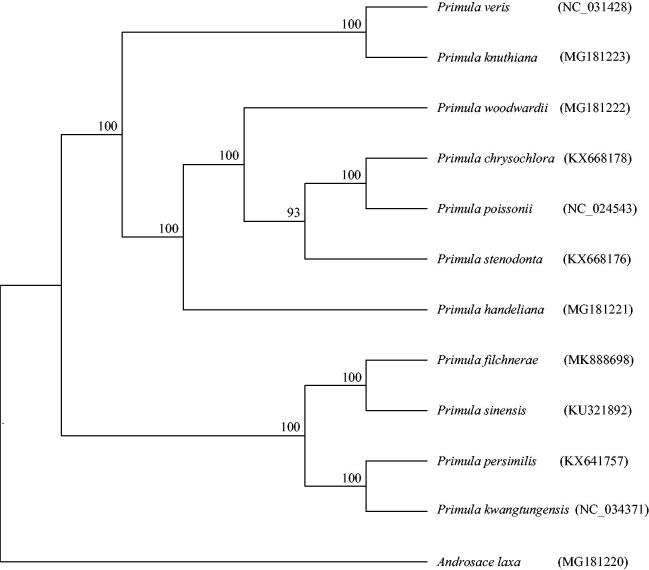
The maximum-likelihood (ML) tree inferred from 12 complete chloroplast genome with 1000 bootstrap replicates. The number on each node indicates the bootstrap value.
